# A controlled study of team-based learning for undergraduate clinical neurology education

**DOI:** 10.1186/1472-6920-11-91

**Published:** 2011-10-30

**Authors:** Nigel CK Tan, Nagaendran Kandiah, Yiong Huak Chan, Thirugnanam Umapathi, Sze Haur Lee, Kevin Tan

**Affiliations:** 1 Office of Neurological Education, Department of Neurology, National Neuroscience Institute, 11 Jalan Tan Tock Seng, Singapore 308433, Singapore; 2 Department of Medicine, Yong Loo Lin School of Medicine, National University of Singapore, 1E Kent Ridge Road, Singapore 119228, Singapore; 3 Department of Biostatistics, Yong Loo Lin School of Medicine, National University Health System, 1E Kent Ridge Road, Singapore 119228, Singapore

## Abstract

**Background:**

Team-based learning (TBL), a new active learning method, has not been reported for neurology education. We aimed to determine if TBL was more effective than passive learning (PL) in improving knowledge outcomes in two key neurology topics - neurological localization and neurological emergencies.

**Methods:**

We conducted a modified crossover study during a nine-week internal medicine posting involving 49 third-year medical undergraduates, using TBL as the active intervention, compared against self-reading as a PL control, for teaching the two topics. Primary outcome was the mean percentage change in test scores immediately after (post-test 1) and 48 hours after TBL (post-test 2), compared to a baseline pre-test. Student engagement was the secondary outcome.

**Results:**

Mean percentage change in scores was greater in the TBL versus the PL group in post-test 1 (8.8% vs 4.3%, p = 0.023) and post-test 2 (11.4% vs 3.4%, p = 0.001). After adjustment for gender and second year examination grades, mean percentage change in scores remained greater in the TBL versus the PL group for post-test 1 (10.3% vs 5.8%, mean difference 4.5%,95% CI 0.7 - 8.3%, p = 0.021) and post-test 2 (13.0% vs 4.9%, mean difference 8.1%,95% CI 3.7 - 12.5%, p = 0.001), indicating further score improvement 48 hours post-TBL. Academically weaker students, identified by poorer examination grades, showed a greater increase in scores with TBL versus strong students (p < 0.02). Measures of engagement were high in the TBL group, suggesting that continued improvements in scores 48 hours post-TBL may result from self-directed learning.

**Conclusions:**

Compared to PL, TBL showed greater improvement in knowledge scores, with continued improvement up to 48 hours later. This effect is larger in academically weaker students. TBL is an effective method for improving knowledge in neurological localization and neurological emergencies in undergraduates.

## Background

"Neurophobia" - a fear of clinical neurology [1]- develops in undergraduates and continues after graduation [2-4]. Students rate their neurology knowledge poorly compared to other medical subspecialties and this may contribute to neurophobia. Many feel the delivery of neurology teaching can be improved [2-4]. To address this knowledge gap, teaching methods employing active learning have been recommended [5,6].

Team-based learning (TBL) is a new educational method that is increasingly used for medical education [7-9]. TBL was originally developed for business education, but has been used for undergraduate [8,10-12] and postgraduate [13,14] medical education. TBL is a teacher-directed method that promotes application of knowledge using small groups in a single venue [15]. It increases learner engagement [11,13,16], promotes active learning, and is perceived as enjoyable by learners [9-11,17]. TBL has been used for a wide range of pre-clinical and clinical subjects [8].

There are few studies comparing TBL to other educational methods. These studies vary in their study designs (randomized trial [13], before-after trials [10,17]) and teaching method comparisons (lectures [13,17,18], tutorials [10]). They also differ in their choice of subjects (undergraduates [10,11,17,18], residents [13]) and controls (same group of students [10,11], randomly selected resident controls [13], or historical controls [12,17,18]). Despite varying methodologies, these studies [10,13,17,19] have demonstrated higher engagement and enjoyment among TBL participants.

There are however conflicting data on whether TBL improves knowledge outcomes compared to other educational techniques. Haidet and colleagues [13] did not find a significant difference in knowledge outcomes between TBL and lectures; another group found improved examination scores in the TBL group but the use of historical controls made interpretation difficult [17]. Another group found improvement in some topics, but not all topics [11]. TBL has not been used in clinical neurology as a teaching tool.

We hypothesized that TBL would be effective for clinical neurology education. We tested our hypothesis by performing a controlled study for undergraduate clinical neurology education comparing TBL to passive learning (PL), measuring knowledge as the primary outcome and self-reported student engagement as a secondary outcome.

## Methods

Our study population comprised third year medical undergraduates from the National University of Singapore. In the third year, students undergo a nine-week posting in internal medicine, where they are rotated into various medical subspecialties including neurology; teaching of all subspecialties runs concurrently. We obtained ethics approval from the institutional ethics committee.

### Selection of intervention and controls

We performed a controlled study of TBL versus PL, using a modified crossover design [11,20]. We selected two topics for TBL: neurological emergencies (NE) and neurological localization (NL). The topics were chosen because NE is a educational priority area [21], while NL is a key topic in the American Academy of Neurology clerkship core curriculum [22] and has been identified as a difficult area for learners [3]. Both these topics may be taught through clinical scenarios which require the participants to make a number of evaluative decisions and judgments en route to selecting a final answer [8].

**Figure 1:**
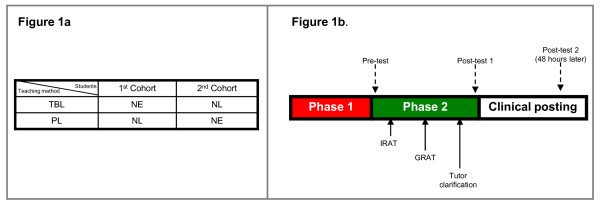
**Allocation of groups to TBL/PL**. TBL: Team-Based Learning, PL: Passive Learning. NE: Neurological Emergencies, NL:Neurological Localization. IRAT: Individual Readiness Assurance Test, GRAT: Group Readiness Assurance Test. **Phases of TBL**. TBL: Team-Based Learning, PL: Passive Learning. NE: Neurological Emergencies, NL:Neurological Localization. IRAT: Individual Readiness Assurance Test, GRAT: Group Readiness Assurance Test.

TBL was administered as the active learning intervention in two cohorts of students. The first cohort was randomly assigned to receive TBL in NE; the second cohort received TBL in NL (Figure 1a). Both cohorts also simultaneously received passive learning (PL) in the other topic that was not covered by TBL. Thus the first cohort acted as the active interventional arm (TBL) in NE while also serving as the control arm (PL) in NL for the second cohort, and vice versa for the second cohort. This design aims for both TBL and PL to be administered simultaneously rather than sequentially as would normally occur in a typical crossover study, avoiding a carryover effect of TBL learning principles to the control group [20].

As we aimed to compare TBL against PL, we have depicted the groups being compared (TBL versus PL) in Figure 1a. Each comparison group consisted of students from both cohorts; each comparison group also included both NE and NL as topics.

### TBL (intervention)

The pure application of TBL [7,8] involves three phases:

Phase 1: Students read preparatory material independently outside of class

Phase 2: Students complete an Individual Readiness Assurance Test (IRAT) to assess their grasp of the knowledge and concepts learned in Phase 1. The same test is then re-administered to the students in pre-assigned groups of 5-7 students; each student was randomly assigned to a group. The team then forms a consensus about each answer in a Group Readiness Assurance Test (GRAT)

Phase 3: Students work in groups on assignments that allow them to apply knowledge gleaned in Phases 1 and 2 to clinical problems.

The TBL method allows flexibility on the part of the teachers to selectively use one or more of the phases, depending on the context and demands of the course. Given the constraints of medical education, such flexibility has previously been exercised [10,15,17,23]. We performed a modified TBL focusing on Phases 1 and 2 (Figure 1b).

We first prepared the students by meeting them to explain the purpose and learning objectives of TBL [24]. The students were then given lecture notes one week before the scheduled TBL and asked to read in preparation for Phase 2.

Phase 2 was conducted in a conference room over 2 hours (Figure 1b) with two of the authors (NCKT, KT) as facilitators. The students first performed the IRAT on clinical scenarios with four or five plausible choices, using an electronic audience response system [25] (ARS) (eInstruction, Denton, Texas, USA). The questions required students to recall facts or concepts learned in Phase 1, and apply this knowledge to derive the correct answer. Six scenarios were used for NE IRAT; seven for NL IRAT.

After IRAT, the class formed into 4 to 5-student teams for GRAT; students were randomly assigned to teams [26]. Each team went through the same clinical scenarios simultaneously, adhering to the '4S' principles [24]. They had three minutes for group discussion and to formulate a team answer by consensus. After group discussion, teams had to simultaneously display their consensus answer on a card; each team could thus see all other teams' choices. The teams had to discuss and justify their answers, with the facilitators clarifying concepts or misconceptions. Teams were allowed to appeal if they felt they had a valid point. Finally, the correct answer was provided, team scores were tabulated, and teams moved to the next scenario.

### Passive learning (control)

Students undergoing the PL topic were also provided lectures notes on the topic at TBL Phase 1 and told to do advance reading. However, the students were unaware which topic (NE or NL) would be used for the TBL Phase 2 until the start of IRAT. By doing so, we aimed to ensure that they read equally diligently for both topics before TBL Phase 2. Prior knowledge of the TBL topic may introduce bias as the students may opt to read the TBL topic more diligently.

### Outcome measurement

We performed three closed-book tests to assess knowledge (Figure 1b) as our primary outcome. Baseline 'pre-test' was administered just before IRAT at Phase 2; the second test ('post-test 1') was done after completion of GRAT at the end of Phase 2; the third test ('post-test 2') was done two days after TBL; this timing was chosen as knowledge attrition occurs three days after passive learning [27].

Each test consisted of 40 true/false questions, covering 10 clinical scenarios (five about NL; five about NE). Questions were designed such that the correct answers required both recall and application of Phase 1 knowledge. Post-tests 1 and 2 consisted of the same scenarios as the pre-test, but with the order of scenarios and questions randomly scrambled. The maximum score was 20 each for NE and NL sections. Students were allotted 20 minutes for each test.

For the primary outcome, we measured the change between each student's post-test score (both post-test 1 and 2) and the baseline pre-test score, and expressed it as a percentage of the total score for that section (NE or NL). For a student who scored 10/20 in the NL section of the pre-test, then 12/20 for post-test 1, and 15/20 for post-test 2, the outcomes would be represented as: 0% (baseline), +10% (post-test 1), +25% (post-test 2).

We measured self-reported student engagement as a secondary outcome, using a modified version of a validated tool [28]. Engagement was measured anonymously using a five-point Likert scale (1 = strongly disagree, 5 = strongly agree). We also measured the proportion of correct IRAT & GRAT answers during Phase 2.

We collected data on students' gender and their second year examination grades. Students who failed (< 50% of maximum score) were graded 'F' by the university. Students who passed (50-100% of maximum score) were graded from 'A' to 'D' by quartiles ('A' 87.5-100.0%, 'D' 50.0-67.4% of maximum score); there was no 'E' grade. We used examination grade as a proxy measurement of each student's baseline medical knowledge.

### Blinding

The students were blinded to the nature, number and timing of the pre-post tests. They were also blinded to the TBL topic until Phase 2. Between post-test 1 and 2, students underwent teaching in internal medicine by non-neurology tutors who were unaware of our study.

### Statistical analysis

Differences in proportions between TBL and PL groups were tested using the χ^2 ^test; differences in means were tested using two-sample *t*-test if normality & homogeneity assumptions were satisfied otherwise the non-parametric Mann-Whitney-U test was applied. A mixed model ANCOVA (analysis of covariance) was performed, taking into account that each student was used twice, to determine differences between TBL & PL groups in the primary outcome, adjusting for gender and examination grades. We also looked for interaction between TBL and gender, TBL and topic, and TBL and examination grades; interaction effects were deemed significant if p < 0.05. Statistical evaluations were performed using SPSS 17.0 based on 2-sided tests; p < 0.05 was considered as significant.

## Results

Forty-nine students participated in our study (first cohort, n = 24; second cohort, n = 25) (table 1). No student declined participation. Mean age was 21.4 ± 0.1 years (all values expressed as mean ± SEM); 55.1% were male. The proportion of correct answers was significantly higher during GRAT compared to IRAT (GRAT 62/78, 79.5% vs IRAT 207/332, 62.3%; p = 0.006).

**Table 1:** Baseline characteristics of students and results of tests

	TBL (n = 49)	PL (n = 49)	P value
Age in years, mean (SEM)	21.4 (0.1)	21.4 (0.1)	1.0

Male, n (%)	27 (55.1)	27 (55.1)	1.0

Examination grades, n (%)			

A	8 (16.3)	8 (16.3)	

B	29 (59.2)	29 (59.2)	

C	11 (22.4)	11 (22.4)	1.0

D	1 (2.0)	1 (2.0)	

F	0 (0.0)	0 (0.0)	

Unadjusted test scores, mean (SEM)			

Pre-test	14.90 (0.30)	14.20 (0.34)	0.13

Post-test 1	16.65 (0.33)	15.06 (0.34)	0.01

Post-test 2	17.18 (0.32)	14.88 (0.29)	< 0.001

Unadjusted mean % change in score from baseline pre-test (SEM)			

Post-test 1	8.8 (1.5)	4.3 (1.3)	0.023

Post-test 2	11.4 (1.6)	3.4 (1.7)	0.001

Students scoring ≥ 90%, n (%)			

Pre-test	5 (10.2)	6 (12.2)	1.0

Post-test 1	20 (40.8)	8 (16.3)	0.013^a^

Post-test 2	26 (53.1)	5 (10.2)	< 0.001^b^

TBL = team based learning

PL = passive learning

SEM = standard error of the mean

^a ^p = 0.009 after adjustment for gender and examination grades

^b ^p < 0.001 after adjustment for gender and examination grades

Complete data for all three pre-post tests were obtained. Mean baseline pre-test scores in both groups were not significantly different (p = 0.13); these scores were not significantly different in males compared to females (p = 0.71), but was higher in those who had examination grades of A or B, compared to those who were graded C or D (15.03 ± 0.25 vs 13.08 ± 0.44, p < 0.001).

After TBL, the unadjusted mean scores for both post-test 1 and post-test 2 increased in both groups; however, the increase was significantly greater in the TBL group. The proportion of students getting ≥ 90% of the questions correct was significantly higher in the TBL versus the PL group.

**Figure 2:**
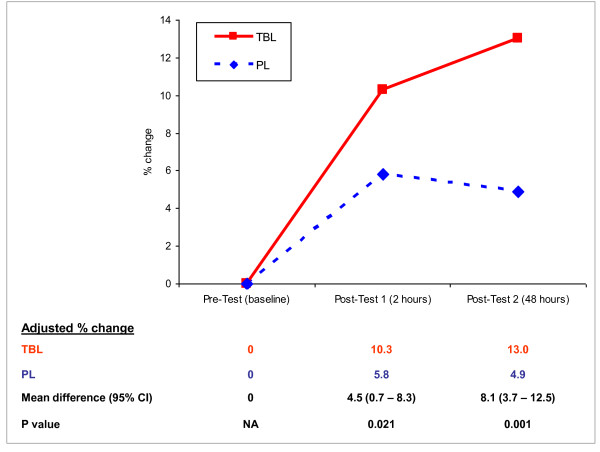
**Mean percentage change in test scores, adjusted for gender and second year examination grades**.

After adjusting for gender and examination grades (Figure 2), for post-test 1, the mean percentage change in the TBL group test scores was significantly greater than in the PL group (10.3% vs 5.8%, mean difference 4.5%, 95%CI 0.7--8.3%, p = 0.021). For post-test 2, the mean percentage change in the TBL group was even greater than in the PL group (13.0% vs 4.9%, mean difference 8.1%, 95%CI 3.7--12.5%, p = 0.001). In the TBL group, test scores increased by the end of TBL and further increased 48 hours later despite no additional teaching in that topic; in contrast, the PL group scores peaked then dropped within 48 hours.

**Figure 3:**
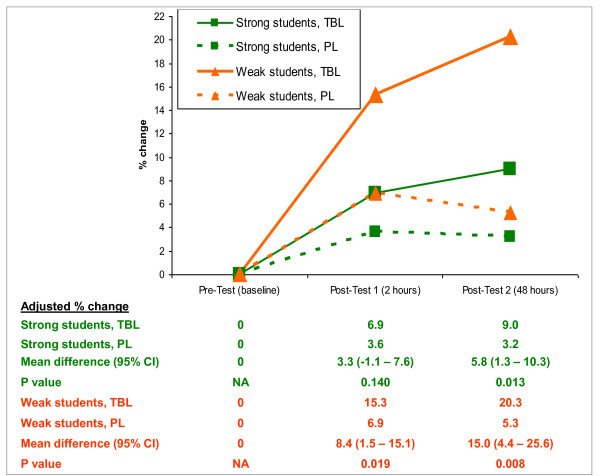
**Adjusted mean percentage change in test scores, strong vs weak students**.

We found no significant interaction of gender or TBL topic with the effect of TBL on the primary outcome. However, the effect of examination grades on improvement in test scores was significant from baseline to post-test 1 (p = 0.007), but was not significant from baseline to post-test 2 (p = 0.22). We dichotomized the students into 'strong' (A-B examination grades) versus 'weak' (C-D grades) students. In both strong and weak students, the TBL group showed a larger increase in the primary outcome compared to the PL group (Figure 3). The effect size however was greater in the weak students for both post-tests (p < 0.02).

Measures of engagement were high in the TBL group (table 2); most students reported active participation in themselves and their peers. The majority (81.6%) preferred TBL to conventional tutorials.

**Table 2:** Measures of engagement in TBL group (n = 49)

Statement	Percentage responding as 'agree' or 'strongly agree'	Median Score*
I actively participated in discussion today	79.6	4

I was mostly an active learner	79.6	4

Most students were actively involved	79.6	4

I had a chance to share my answers, or have my questions answered	85.7	4

TBL is more enjoyable than conventional teaching	93.9	5

* scores rated on a 5-point Likert scale (1 = strongly disagree, 5 = strongly agree).

## Discussion

We used a novel study design to explore the effect of TBL as an active learning method compared to PL, measuring knowledge outcomes immediately and 48 hours after intervention. Students receiving PL showed initial improved scores, which fell 48 hours later. Conversely, scores increased immediately after TBL and increased further 48 hours later. In addition, this effect of TBL is larger in academically weaker students compared to strong students.

An earlier randomized controlled trial [13] comparing TBL against conventional lectures among residents did not demonstrate superiority in knowledge outcomes, possibly due to loss to follow-up or learner heterogeneity. Another group found improvement in some, but not all topics [11]. Our study had complete follow-up data, and involved students from the same academic year, which may have allowed us to demonstrate superiority of TBL over PL.

Our finding that TBL improves knowledge immediately compared to PL is not surprising, as TBL has been shown to reinforce concepts and aid application [7]. TBL utilizes the active processing principle [29], making learners solve a clinically relevant problem by using prior knowledge, thus creating meaningful learning. Constructivist theories may also come into play, especially when teachers act as facilitators to aid learning [30]. Success in answering GRAT questions correctly may possibly raise self-efficacy [30]. These learning theories might explain the immediate improvement in knowledge.

However, the additional improvement 48 hours later was striking, particularly when compared to the score decrease in the PL group. This interesting finding leads us to hypothesize that TBL encouraged self-directed learning [30,31] via peer discussion, self-reflection or further self-reading, allowing learners to reinforce and retain knowledge. Alternatively, TBL may stimulate higher-level thinking skills or improve long-term ability to recall and use concepts and strategies [29,32] for problem solving, thus protecting against knowledge attrition. The improvement in post-test 2 for the TBL group is unlikely to be due to the practice effect, as this should have led to improved scores for both groups with repeated testing. The practice effect was also mitigated by randomly scrambling the order of questions in all the tests. Our findings are also unlikely to be due to students deliberately preparing for the TBL topic for post-test 2, as they were unaware of the nature and timing of the tests. Our results accord with that of another study showing that knowledge is better retained after TBL, though the duration of retention was longer (19 weeks) in that study [18].

During the GRAT, we found enthusiastic student participation and interaction. The students also reported high levels of engagement by themselves and the class, consistent with previous TBL studies [9,10,13,15,17,19,33]; 81.6% found TBL to be more enjoyable than conventional tutorials. This high level of engagement may have contributed to self-directed learning [30] after the end of TBL [31,32] explaining the additional improvements in post-test 2 scores, and thus the superiority of TBL over PL, as there was no student interaction during PL. Further studies could perhaps compare TBL against other active learning methods such as problem-based learning or small-group learning.

Our finding that TBL has a greater effect in academically weaker students is consistent with extant literature [18,34]. We believe that this finding has relevance to neurology education and the problem of neurophobia [1]. 'Neurophobic' students lack neurology knowledge [2,3]. TBL may thus be able to help weaker 'neurophobic' undergraduates gain and reinforce knowledge compared to passive learning methods.

The strengths of our study include learner homogeneity, use of a PL comparator, matching of TBL and PL groups and mitigation of the carryover effect using our study design, blinding of students to study design and testing, relevance of topics [21,22], measures taken to minimize the practice effect, delayed post-testing 48 hours later, and complete follow-up.

We selected self-reading as the PL comparator, although previous studies have compared TBL with lectures [13,18], tutorials [10] or small-group learning [11]. There is no consensus as to what would be a suitable comparator for studies assessing an active learning method; both passive [10,13,18] and active [11] methods have been used. Similar to a clinical trial, our treatment (TBL) should be compared to a placebo to show efficacy. For ethical reasons, we cannot use a placebo as it would be tantamount to not teaching students a topic. We therefore selected reading as the PL based on prior studies that showed reading as the most passive learning method [27]. Using reading as a PL comparator allows the control students a way to learn, thereby fulfilling ethical imperatives, while methodologically serving as a fair comparator for TBL. After study completion, each student cohort also received a didactic lecture on their PL topic to reinforce learning.

Our study has several limitations. Firstly, our sample size is small as only 49 students were involved; a larger study would be helpful to confirm our findings. Secondly, post-test 2 was similar to post-test 1; new questions added to post-test 2 may have allowed learners to apply newly-obtained knowledge to fresh questions. Our tests therefore predominantly assess retention of knowledge at 48 hours, rather than the ability to apply knowledge to new clinical scenarios at the end of their nine-week posting. True/false questions also have inherent limitations [35].

Thirdly, due to time constraints in a busy nine-week internal medicine posting, we conducted a modified TBL focusing on Phases 1 and 2 instead of a full programme [7]. However, as prior medical TBL studies have also performed similarly modified TBL, such modifications may be sufficiently effective in medical education [10,13,18,23]. In our TBL implementation we adhered wherever possible to core TBL principles by using a scorecard [26] and the '4S' principles [24]. Some authors are concerned that partial TBL implementations may lead to negative conclusions about the efficacy of TBL [36]. Despite a modified TBL programme, we found TBL superior to PL; a full implementation may have shown an even greater effect. Finally, our finding of a larger effect in weak students is based on a subgroup analysis [37]. This finding needs corroboration by further studies, but the *a priori *defined subgroup, confirmation with formal testing for interaction, statistical significance and lack of multiple subgroup testing [38] in our study suggest that this may be a true effect.

## Conclusion

TBL improves knowledge scores in undergraduate neurology education, with sustained and continuing improvement up to 48 hours later. This effect is greater in academically weaker students. Students taught by TBL report high engagement which may promote greater self-directed learning. With increasing emphasis on active learning in neurology [5], our results suggest TBL may be a useful adjunct teaching method for undergraduate neurology education, particularly for academically weaker learners.

## List of abbreviations

ANCOVA: Analysis of Covariance; ARS: Audience Response System; GRAT: Group Readiness Assurance Test; IRAT: Individual Readiness Assurance Test; NE: Neurological emergencies; NL: Neurological localization; PL: Passive learning; TBL: Team-based learning.

## Competing interests

The authors declare that they have no competing interests.

## Authors' contributions

NCK Tan had full access to all the data in the study and takes responsibility for the integrity of the data and the accuracy of the data analysis. Study conception and design: NCKT, KT, NK. Acquisition of data: NCKT, KT, SHL, TU. Analysis and interpretation of data: NCKT, YHC, KT, NK. Drafting of manuscript: NCKT, YHC. Critical revision of manuscript for important intellectual content, and final approval of manuscript: All authors

## Pre-publication history

The pre-publication history for this paper can be accessed here:

http://www.biomedcentral.com/1472-6920/11/91/prepub
